# The unique hypertrophic and fibrotic features of neonatal right ventricle in response to pressure overload

**DOI:** 10.1038/s41598-025-01427-y

**Published:** 2025-05-20

**Authors:** Yingying Xiao, Yiting Xue, Debao Li, Lincai Ye, Zheng Wang, Sixie Zheng, Peisen Ruan, Hao Chen, Haifa Hong

**Affiliations:** 1 Department of Thoracic and Cardiovascular Surgery, Shanghai Children’s Hospital, Shanghai Jiao Tong University School of Medicine, Shanghai, China; 2 Department of Thoracic and Cardiovascular Surgery, Shanghai Children’s Medical Center, Shanghai Jiao Tong University School of Medicine, Shanghai, China; 3 Department of Pediatric Surgery, Children’s Hospital of Fudan University, National Children’s Medical Center, Shanghai, China; 4 Institute of Pediatric Translational Medicine, Shanghai Children’s Medical Center, Shanghai Jiao Tong University School of Medicine, Shanghai, China; 5 Shanghai Institute for Pediatric Congenital Heart Disease, Shanghai Children’s Medical Center, Shanghai Jiao Tong University School of Medicine, Shanghai, China; 6 Department of Critical Care Medicine, The Affiliated Women and Children’s Hospital of Ningbo University, Ningbo, Zhejiang China

**Keywords:** Pressure overload, Right heart failure, Pediatric, Single-cell sequencing, Right ventricle, Cardiovascular biology, Cardiovascular diseases

## Abstract

Pediatric heart failure (HF) research remains in its infancy partly due to the lack of neonatal rat/mouse models of HF. The aim of the study is to introduce a neonatal rat/mouse model of right ventricular (RV) pressure overload (RVPO), a significant cause of pediatric HF, and to uncover the molecular features of RVPO-induced RV hypertrophy and fibrosis—the two most important transitional pathological states between normal and dysfunctional RV. Neonatal rat/mouse model of RVPO was established by pulmonary artery banding (PAB) surgery on postnatal day 1(P1) and confirmed by echocardiography and morphological examination on P7. Bulk RNA and single-cell RNA sequencing was performed on RV tissues, along with bulk RNA sequencing on RV cardiomyocytes, to screen a range of key genes and signaling pathways that are upregulated and that play critical roles in adult hypertrophy and fibrosis. The sequencing results were further verified by qRT-PCR and histological staining. Most of the pathways and associated genes, such as oxidative stress, inflammation, phosphodiesterase, proteasome, protein kinase, transforming growth factor, and angiotensin were not changed or downregulated in the neonatal RVPO model. This study reveals the unique features of hypertrophy and fibrosis in the neonatal RV in response to pressure overload, which partly explains why adult-effective anti-HF drugs fail to treat pediatric HF. More importantly, single-cell RNA sequencing data of the neonatal RV with pressure overload were documented, providing an important reference for future basic or clinical investigations on pediatric RV failure.

**Supplementary Information:**

The online version contains supplementary material available at 10.1038/s41598-025-01427-y.

## Introduction

Pediatric HF is a fatal disease, and its mortality rate exceeds that of many common pediatric cancers^1–3^; it is therefore a serious burden to the society and families^1,2^, although considerably less in absolute terms compared with adult HF. However, the death rates and resources required per patient in pediatric HF are higher than those in adults with HF^1,2^. More importantly, most pediatric HFs are caused by CHD. For example, research has shown that 47% of emergency department visits and 63% of hospitalizations for pediatric HF were concomitant with CHD^1,2^, and 15% of pediatric CHD patients who received single ventricle reconstruction developed HF, and 50% of those patients died of HF^4^. As a result, CHD is the most common indication for pediatric heart transplantation^5^.

However, pediatric HF research remains in its infancy^1–3^. Current treatment protocols, drugs, and targets for pediatric HF are derived from adult HF research, thus failing to improve the quality of life of children with HF^6–9^. One of the possible reasons for this situation is the lack of neonatal CHD rat/mouse models^10,11^, which requires skilled and challenging microsurgery. Right ventricular pressure overload (RVPO), one of the most important features of various types of CHD, such as pulmonary artery stenosis, Tetralogy of Fallot (TOF), and pulmonary vein stenosis, and pulmonary arterial hypertension (PAH), usually determine the prognosis and surgical approach of pediatric patients^10–12^. Thus, further research is needed to understand the molecular features of RVPO-induced pediatric HF, and this was one of the aims of this study.

In adults, RVPO usually leads to RV hypertrophy and fibrosis—the two most important transitional pathological states between normal and dysfunctional RV—have been extensively studied^13,14^. Multiple pathways, such as oxidative stress, inflammation, phosphodiesterase, proteasome, protein kinase, transforming growth factor, and angiotensin, have been shown to be activated/upregulated in adult RVPO animal models and have been used to design targets for anti-hypertrophy or anti-fibrosis^15–23^. However, due to the lack of neonatal rat/mouse models with RVPO, the molecular features of hypertrophy and fibrosis of the neonatal RV in response to RVPO are largely unknown.

In addition, clinically, many adult heart failure (HF) treatment-effective drugs failed to improve pediatric HF^24–31^, and most treatment recommendations for patients with a failing RV are based on data from retrospective studies and expert opinion^25,26^. For example, sildenafil and tadalafil, the inhibitor of phosphodiesterase 5 (PED5), which was upregulated in adult RVPO models^17^, have been the first-line drugs to treat PAH for over 10 years^25–27^. A recent, double-blind, randomized, placebo-controlled, multicenter superiority trial (NCT03049540) involving 100 adults with systemic RV failed to show any improvement in RV function, exercise capacity, and neuro-hormonal activation with the treatment of sildenafil for 3 years^26^. Enalapril, an angiotensin-converting enzyme inhibitor, is highly effective in treating adult HF^28,29^; yet, it failed to treat pediatric HF^7,9^. These studies highlight the need for a full understanding of the molecular features of hypertrophy and fibrosis of neonatal RV in response to RVPO.

In 2017, we reported the first neonatal rat model of RVPO achieved by pulmonary artery banding (PAB)^30^. Using this model, it was demonstrated that RVPO improved neonatal cardiomyocyte proliferation in rats^31^, and this was confirmed by a series of subsequent studies^32–34^. These findings corrected the misunderstanding of cardiomyocyte proliferation in children with Tetralogy of Fallot (TOF), characterized by RVPO, as reported in the *New England Journal of Medicine* and *Science Translational Medicine*, which suggested reduced cardiomyocyte proliferation in children with TOF^35,36^. After several years of research, in 2024, the first neonatal mouse model of RVPO was reported, demonstrating that RVPO improved neonatal cardiomyocyte proliferation in mice^11^. It was also found that RVPO induced neonatal RV hypertrophy but not fibrosis both in rats and mice in terms of histology^11,31^. However, the molecular features of hypertrophy and fibrosis of neonatal RV in response to RVPO have not yet been reported, and it remains to be determined whether they respond differently from those in adult RV in response to RVPO. In this study, bulk RNA and single-cell RNA sequencing was used in combination with other techniques to determine the molecular features of hypertrophy and fibrosis of neonatal RV in response to RVPO at the transcriptional levels.

## Materials and methods

### Data availability

All of the bulk RNA-seq data were deposited in the GEO database (https://www.ncbi.nlm.nih.gov/geo) with accession numbers GSE139561 and GSE232054. Single-cell RNA-seq data are available from the corresponding author upon reasonable request.

Information on all primers is provided in Supplemental Tables S1.

### Ethical statement

This study is performed in accordance with relevant guidelines and regulations. All methods are reported in accordance with ARRIVE guidelines, and were approved by the Animal Welfare and Human Studies Committee at Shanghai Children’s Medical Center, China (IRB no: SCMCIRB-K2022146-1).

### Neonatal RVPO animal model construction

Pregnant Sprague–Dawley rats and C57/BL6 mice were purchased from Jihui Experimental Animal Co., Ltd (Shanghai, China). PAB or sham surgery was performed on postnatal day 1 (P1) according to methods outlined in previous studies to generate neonatal RVPO^11,31^. Briefly, after being anesthetized by ice cooling, the neonates (male or female) were transferred to an ice bed and fixed in the supine position, and the pulmonary artery (PA) was exposed by thoracotomy. The PA and the padding needle (28-gauge for rats and 30-gauge for mice) were then tied together using an 11 − 0 nylon thread. After removing the padding needle, a fixed, constricted PA lumen was achieved. Finally, the thoracic wall was closed, and the neonates were warmed and returned to their mothers.

### Histology

At P7, the neonates were sacrificed and their hearts were harvested for hematoxylin and eosin (H&E) staining, Masson staining, and immunofluorescence staining. H&E or Masson staining was performed with an H&E staining kit (C0105M, Beyotime Biotech, Shanghai, China) or a Masson’s Staining kit (C0189S, Beyotime), respectively, according to the manufacturer’s instructions.

For immunofluorescence analysis, the paraffin-embedded heart sections were dewaxed and rehydrated, and the antigen was retrieved. Then, the sections were blocked (PBS with 7.5% goat serum and 0.5% Triton X-100) and subsequently incubated with anti-cardiac troponin T (cTnT, 8295, Abcam, Cambridge, UK, dilution, 1:200) overnight at 4 °C. The next day, the sections were incubated with Alexa Fluor 488 secondary antibodies (ab150077, ab150078, Abcam, Cambridge, UK, dilution, 1:500) at room temperature. After washing, the sections were incubated with 4’,6-diamidino-2-phenylindole (DAPI, C1005) and Alexa Fluor™ 555-labeled wheat germ agglutinin (WGA) (W32466, Thermo Fisher Scientific Inc., Waltham, USA, dilution: 1:100). Finally, the sections were mounted with an anti-quenching resident medium and sealed with nail polish.

### Transthoracic echocardiography

At P7, P14, and P30, the mice or rats were anesthetized with 1.5–2.0% isoflurane. Echocardiograms were analyzed with a Vevo 2100 imaging system (Visual Sonics, Toronto, Ontario, Canada), and a long-axis view of the PA was used to measure the blood flow patterns across the PA constriction by continuous wave Doppler.

### Cardiomyocyte isolation and purification

At P7, the young rats were decapitated. The hearts were removed carefully from the chest, and the hearts were dissociated by Langendorff reverse coronary perfusion with perfusion buffer (135 mM NaCl, 5.4 mM KCl, 0.33 mM NaH2PO4, 1 mM MgCl2, 10 mM HEPES, 10 mM glucose, 5 mM Taurine, 10 mM 2,3-butanedione monoxime (BDM), 200 U/ml II-Collagenase) at 37 °C for 20 min. After perfusion, the RV free wall was resected under a microscope; and RV cardiomyocytes were dissociated by gentle washing, enriched by differential adherence and low speed centrifugation. The purity of the cardiomyocytes was confirmed by flow cytometry. The purified cardiomyocytes were used for bulk RNA-seq and real-time quantitative PCR analysis.

### Real-time quantitative PCR analysis

At P7, mouse RV tissues or rat purified cardiomyocytes were subjected to mRNA extraction using a PureLink RNA Micro Scale Kit (Catalog No. 12183016; Life Technologies, Carlsbad, CA, USA). Reverse transcription-polymerase chain reaction (RT-PCR) was performed using the PrimeScript™ reagent kit (Takara Bio, Kusatsu, Japan). Quantitative real-time polymerase chain reaction (qRT-PCR) was carried out using SYBR Green Power Premix Kits (Applied Biosystems, Foster City, CA, USA) according to the manufacturer’s instructions.

### Bulk RNA-seq

The aforementioned mRNAs were used for the preparation of sequencing libraries using the NEBNext^®^ Ultra™ RNA Library Prep Kit for Illumina^®^ (NEB, USA) following the manufacturer’s recommendations. Library quality was assessed on an Agilent Bioanalyzer 2100 system. The library preparations were sequenced on an Illumina Novaseq 6000 platform. Raw data (raw reads) in fastq format were processed through in-house Perl scripts to generate clean data (clean reads). All of the downstream analyses were based on the clean data. The Fragments Per Kilobase of transcript sequence per Million base pairs sequenced (FPKM) value of each gene was calculated with featureCounts v1.5.0-p3. Differential expression analysis was performed using the DESeq2 R package (1.16.1). Genes with *P* < 0.05 found by DESeq2 were assigned as differentially expressed. Gene Ontology (GO) enrichment analysis of differentially expressed genes (DEGs) was implemented by the clusterProfiler R package, and GO terms with corrected P-values ˂ 0.05 were considered to be significantly enriched by DEGs.

### Single-cell RNA-seq

At P7, the rat RV tissues (two sham and two PAB) were harvested and lysed in chilled Nuclei EZ Lysis Buffer. After washing, the nuclei were filtered through a 35-µm cell strainer. The integrity and number of nuclei were determined by microscopy. Finally, the nuclei were processed using the standard 10X Genomics single-cell protocol. The analyses were performed on the Novogene platform (https://magic.novogene.com) with the R package provided by Novogene Biotechnology Corp (Beijing, China).

### Statistical analysis

Continuous data, including mRNA expression, protein expression, and number of Ki67/pHH3/ aurora B-positive cells, were expressed as means ± standard deviation.

Student’s *t*-test was used to determine whether the data were normally distributed; otherwise, they were tested with the rank sum test. P-values < 0.05 were considered statistically significant. Statistical analyses were performed using SAS software version 9.2 (SAS Institute Inc., Cary, NC, USA).

## Results

### RVPO induces RV hypertrophy

**Fig. 1:**
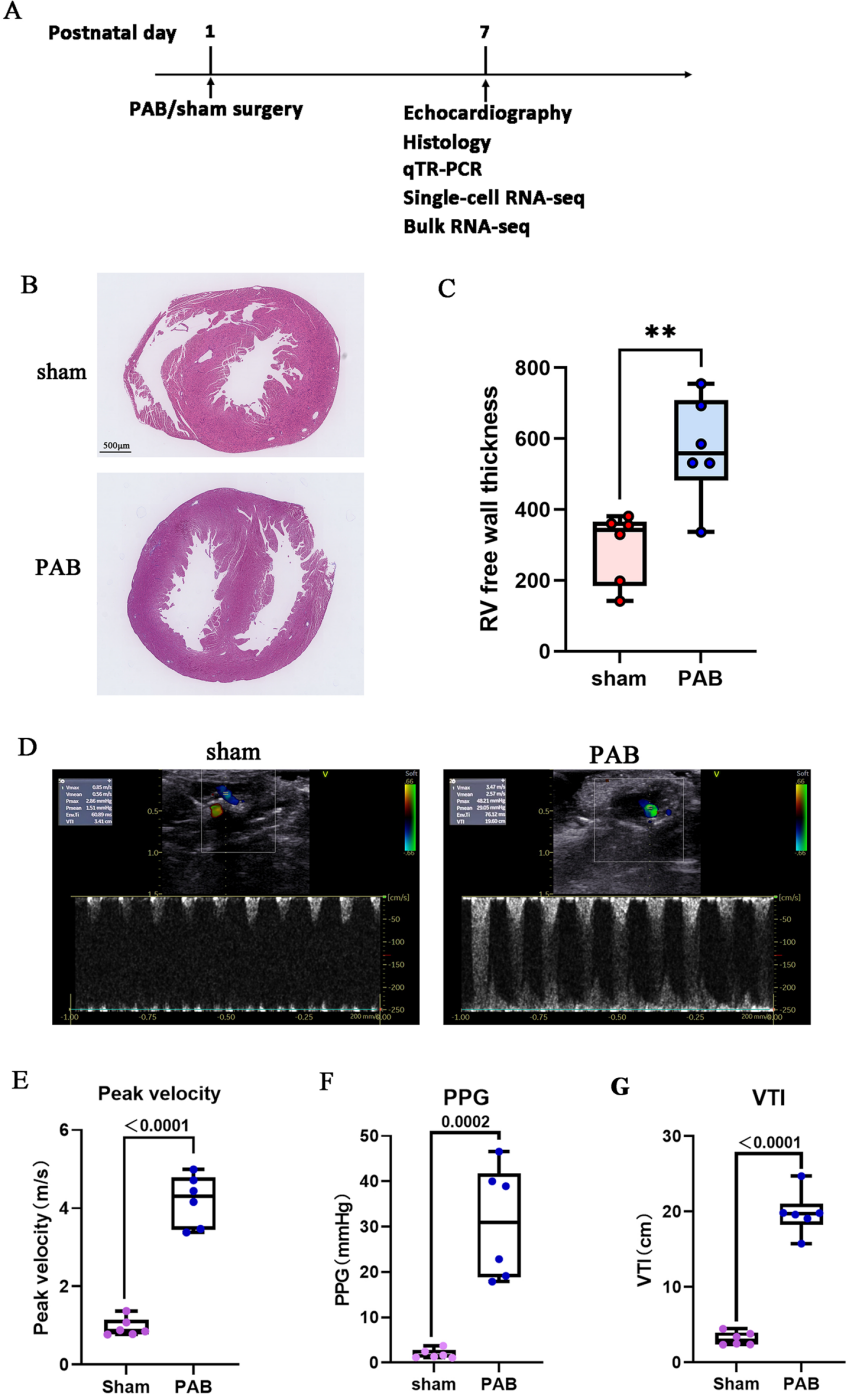
Right ventricular pressuure overload (RVPO) induces RV hypertrophy. (**A**) Timeline of experiments in the current study. (**B**) H&E-stained 2-chamber cross sections of P7 mouse hearts after sham and PAB surgery. (**C**) Quantification of RV free wall thickness of P7 hearts after sham and PAB surgery. (**D**) Representative echocardiography from the sham and PAB mice. (**E**) Quantification of peak velocity. (**F**) Quantification of Peak Pressure gradient (PPG). (**G**) Quantification of velocity-time integral (VTI).

As shown in Fig. 1A, PAB surgery was performed at P1, and all examinations were performed at P7. A right-to-left shift of the ventricular septum was observed in the PAB hearts (Fig. 1B) and there was a significantly increase of peak velocity, peak pressure gradient (PPG), and VTI in the PAB than the sham groups(Fig. 1D-G), indicating a notable formation of RVPO in the PAB mice. As a result, RV free wall thickness was significantly increased in the PAB mice when compared with that of sham mice (Figs. 1B and C), which suggests that RVPO induces neonatal RV hypertrophy, similar to previous research^11,30^.

### RVPO induces RV cardiomyocyte hypertrophy but not fibrosis

**Fig. 2:**
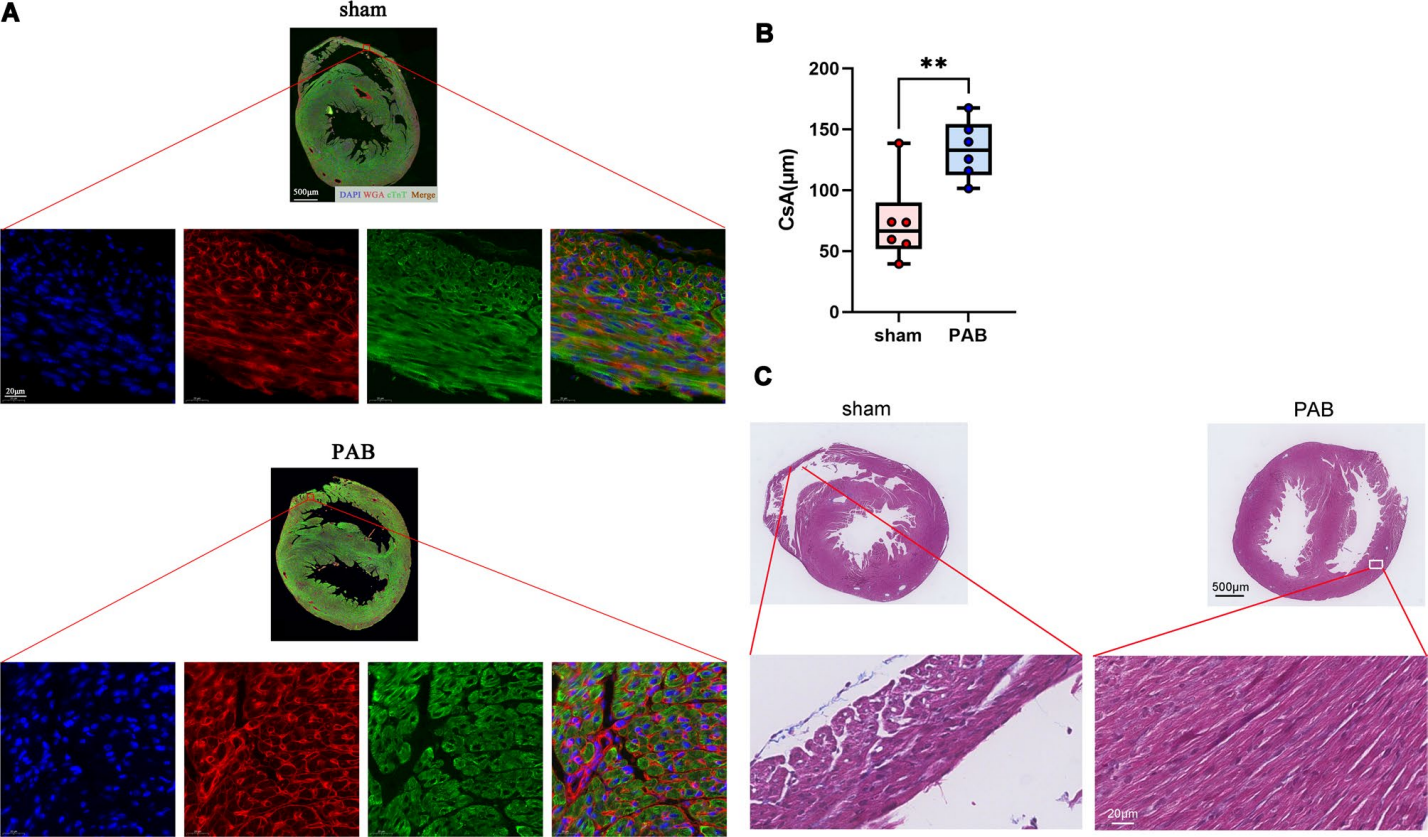
RVPO induces cardiomyocyte hypertrophy but not RV fibrosis. (**A**) Representative cross-sectional area (CsA) of cardiomyocytes of P7 mouse hearts after sham and PAB surgery. WGA (red), cardiac troponin T (cTnT, green), and DAPI (blue). (**B**) Quantification of CsA. (**C**) Representative Masson staining of P7 mouse hearts after sham and PAB surgery.

We then examined the cross-sectional area (CsA) of RV cardiomyocytes. As shown in Figs. 2A and B, the CsA of RV cardiomyocytes was significantly increased in the PAB mice when compared to that of sham mice, suggesting that RVPO induces RV cardiomyocyte hypertrophy. However, we did not find any fibrotic production deposition in the hypertrophic RV (Fig. 2C). These results suggest that RVPO induces neonatal RV cardiomyocyte hypertrophy but not fibrosis.

### A unique hypertrophic and fibrotic feature of neonatal RV tissues in response to RVPO, as revealed by bulk RNA-seq analysis

**Fig. 3:**
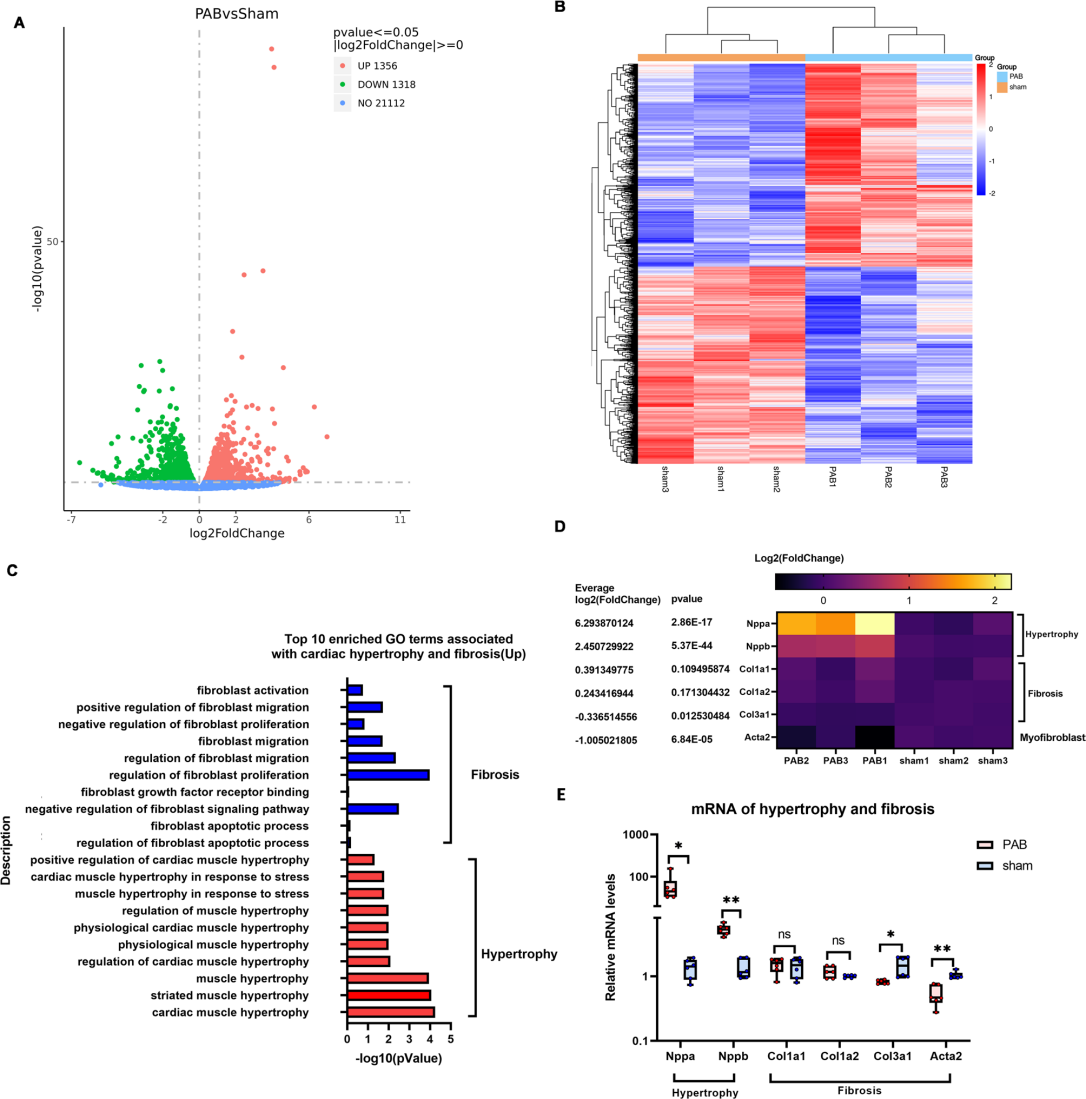
Bulk RNA-seq analysis of RV tissues demonstrates the unique hypertrophic and fibrotic feature of neonatal RV in response to RVPO. (**A**) Volcano plot demonstrates that RVPO generates thousands of DEGs between the sham and PAB groups at P7. (**B**) Cluster heatmap analysis of the DEGs in the sham and PAB groups at P7 reveals similarities within groups and differences between groups. (**C**) Top 10 enriched GO terms associated with cardiac hypertrophy and fibrosis in upregulated DEGs. (**D**) Heatmap of Log2 (fold-change) of hypertrophy and fibrosis marker genes. (**E**) Relative mRNA levels of hypertrophy and fibrosis marker genes. Cluster heatmap was generated using the OECloud tools (v1.26) at https://cloud.oebiotech.com.

To understand the hypertrophic and fibrotic features of neonatal RV tissues in response to RVPO, bulk RNA-seq analysis was performed on RV tissues. The results showed that RVPO generated 2,647 DEGs in the neonatal RV, with 1,356 upregulated DEGs and 1,318 downregulated DEGs (Fig. 3A). Cluster analysis of the DEGs demonstrated that there were a high number of inter-group differences and intra-group consistency (Fig. 3B). GO enrichment analysis of the DEGs revealed abundant enrichment of terms associated with hypertrophy and fibrosis (Fig. 3C). However, fibrosis-associated terms were fibroblast activation, proliferation, or migration, among others, and there were no terms associated with fibrotic production (Fig. 3C). As fibroblasts secrete anti-oxidative, anti-inflammatory, and growth factors—which are important for normal cardiomyocyte function^13,37,38^—it is no surprise to see the enrichment of fibroblast-associated terms.

Consistent with this, a heatmap of log2 (Fold-change) showed that hypertrophy maker genes (NPPA and NPPB) were highly upregulated in the PAB mice, while fibrosis-related maker genes (COL1a1, COL1a2, and COL13a1) and myofibroblast marker (Acta2) showed no changes or were downregulated (Fig. 3D). qPCR results further confirmed that RVPO induced neonatal RV hypertrophy but not fibrosis (Fig. 3E).

**Fig. 4:**
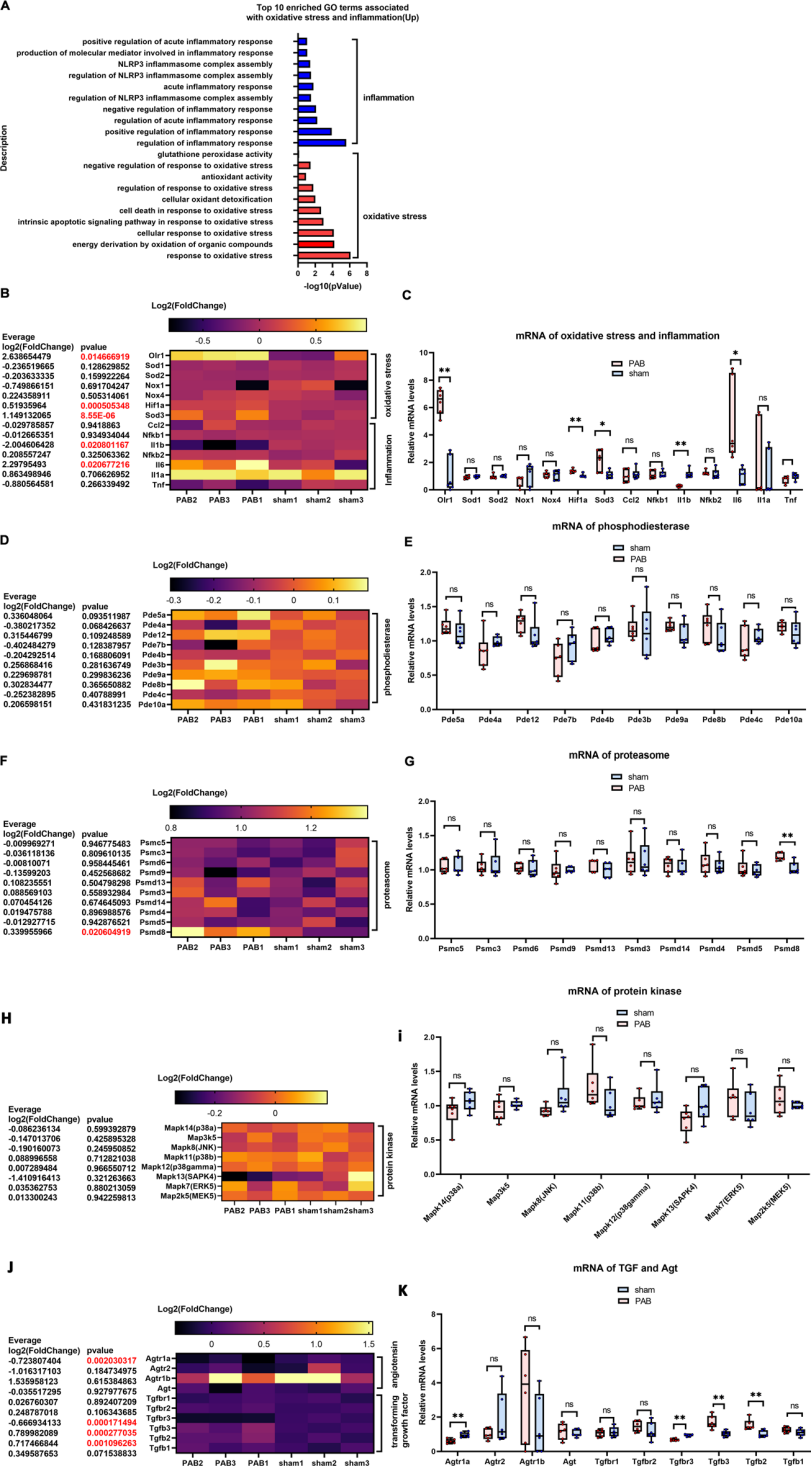
Bulk RNA-seq analysis of RV tissues demonstrates that the signaling pathways that account for hypertrophy and fibrosis in neonatal RV are different from those in adult RV. (**A**) Top 10 enriched GO terms associated with oxidative stress and inflammation in upregulated DEGs. (**B**) Heatmap of Log2 (fold-change) of oxidative stress and inflammation marker genes. (**C**) Relative mRNA levels of oxidative stress and inflammation marker genes. (**D**) Heatmap of Log2 (fold-change) of phosphodiesterase genes. (**E**) Relative mRNA levels of phosphodiesterase genes. (**F**) Heatmap of Log2 (fold-change) of proteasome genes. (**G**) Relative mRNA levels of proteasome genes. (**H**) Heatmap of Log2 (fold-change) of protein kinase genes. (**I**) Relative mRNA levels of protein kinase genes. (**J**) Heatmap of Log2 (fold-change) of TGF and AGT. (**K**) Relative mRNA levels of TGF and AGT genes. Cluster heatmap was generated using the OECloud tools (v1.26) at https://cloud.oebiotech.com.

Studies on adult RVPO animal models have shown that oxidative stress and inflammation are two of the initiators of RV hypertrophy and fibrosis^15,16^. Results of this study found enrichment of GO terms associated with inflammation and oxidative stress but with anti-inflammation and anti-oxidative stress GO terms (Fig. 4A). The reported and important genes in adult RVPO animal models were screened^15–23^, and as shown in Figs. 4B, C, in neonatal PO-RV, the expression of oxidized low-density lipoprotein receptor 1 (OLR1) and interleukin-6 (IL-6), which promote oxidative stress and inflammation, was upregulated and consistent with those in adult PO-RV^39,40^. In contrast to those in adult PO-RV, the expression of HIF-1α and SOD3, which function in anti-oxidative stress and inflammation, was upregulated, and interleukin-1 beta (IL-1b) was downregulated in neonatal PO-RV (Figs. 4B and C). The other key genes, upregulated in adult PO-RV^13,37,38^, such as SOD1, SOD2, NOX1, NOX4, CCl2, NF-kB1, NF-kB2, IL1a, and TNF, were unchanged (Figs. 4B and C).

The key regulators and pathways for hypertrophy and fibrosis in neonatal PO-RV tissues were screened. The expression of all types of phosphodiesterase was unchanged (Figs. 4D and E). In the proteasomes, only the expression of PSMD8 was upregulated (Figs. 4F and G), while there were no changes in the p38-JNK-MEK pathways (Figs. 4H and I). In the angiotensin (AGT) system, the AGTR1a was downregulated, and the other AGTs were unchanged (Figs. 4J and K); TGFB2 and TGFB3 were upregulated, while TGFB3R was downregulated, and the other TGFs were unchanged (Figs. 4J and K).

In summary, bulk RNA-seq analysis of PO-RV tissues revealed a unique hypertrophic and fibrotic feature of neonatal RV, characterized by inflammation and oxidative stress, phosphodiesterases, proteasomes, the p38-JNK-MEK pathways, the AGT system, and the TGF system.

### A unique hypertrophic and fibrotic feature of neonatal RV cardiomyocytes in response to RVPO, as revealed by bulk RNA-seq analysis

**Fig. 5:**
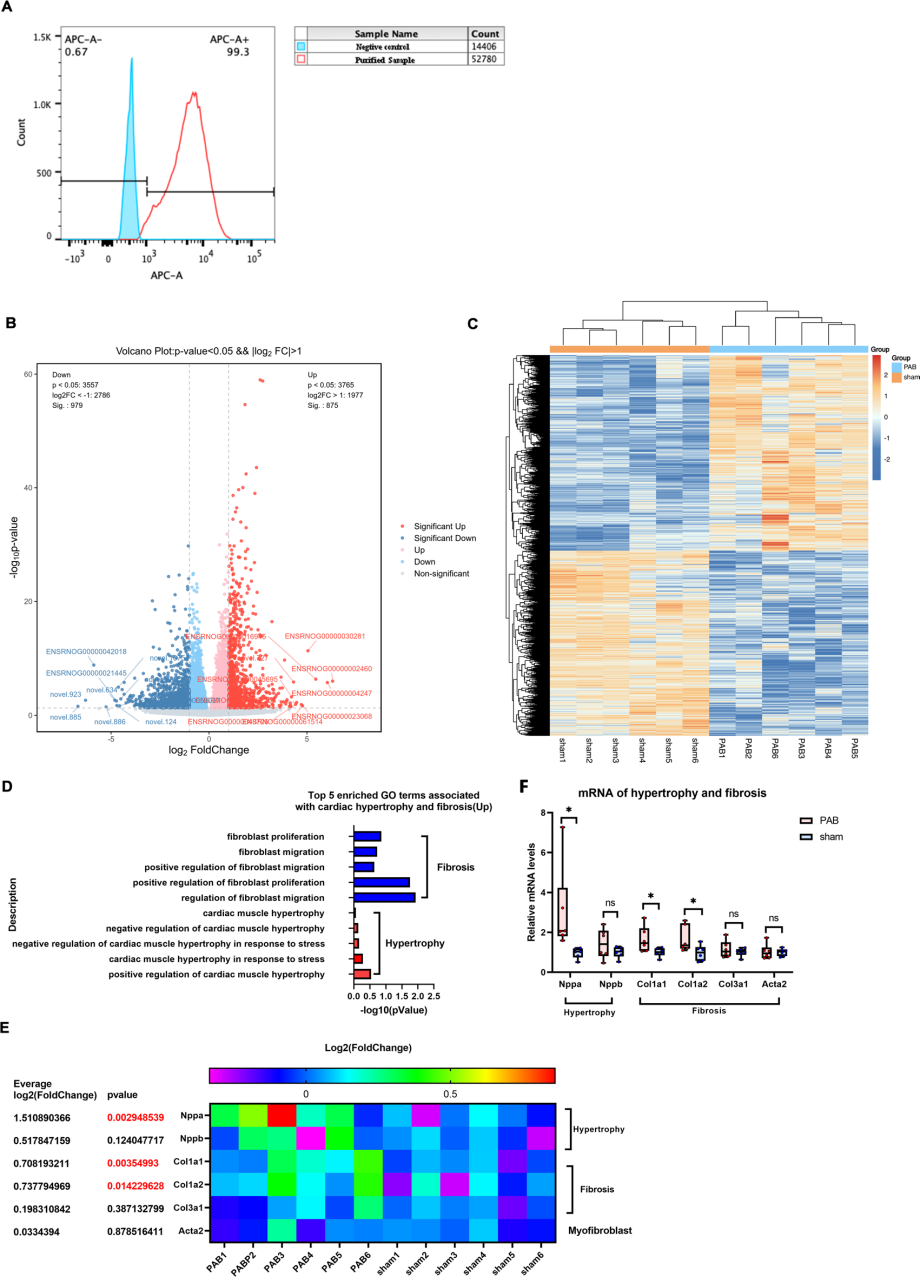
Bulk RNA-seq analysis of RV cardiomyocytes demonstrates the unique hypertrophic and fibrotic feature of neonatal RV in response to RVPO. (**A**) Flow cytometry indicates that ~ 99% of purified cells were cTnT-positive. (Obtained from Ye L, et al. J Am Heart Assoc. 2020;9(11):e015574. with the permission of the publisher). (**B**) Volcano plot demonstrates that RVPO generates thousands of DEGs in cardiomyocytes between the sham and PAB groups at P7. (**C**) Cluster heatmap analysis of the DEGs in cardiomyocytes between the sham and PAB groups at P7 reveals similarities within groups and differences between groups. (**D**) Top 5 enriched GO terms associated with cardiac hypertrophy and fibrosis in upregulated DEGs of cardiomyocytes. (**E**) Heatmap of Log2 (fold-change) of hypertrophy and fibrosis marker genes in cardiomyocytes. (**F**) Relative mRNA levels of hypertrophy and fibrosis marker genes in cardiomyocytes. Cluster heatmap was generated using the OECloud tools (v1.26) at https://cloud.oebiotech.com.

As previously shown, RVPO induced a similar hypertrophy and fibrosis of neonatal RV both in mice and rats^11,31^. PAB surgery was performed on neonatal rats, whose hearts provide significantly more cardiomyocytes than mouse hearts. Cardiomyocytes were isolated and purified (Fig. 5A) for bulk RNA-seq analysis to understand the hypertrophic and fibrotic features of neonatal RV cardiomyocytes in response to RVPO and to further confirm the RNA-seq results of the RV tissues. The results showed that RVPO generated 7,322 DEGs in the neonatal RV cardiomyocytes, with 3,765 upregulated DEGs and 3,557 downregulated DEGs (Fig. 5B). Cluster analysis of the DEGs demonstrated that there were a high number of inter-group differences and intra-group consistencies (Fig. 5C). GO enrichment analysis of the DEGs showed that there was also enrichment of terms associated with hypertrophy and fibrosis in cardiomyocytes (Fig. 5D), as seen in RV tissues (Fig. 3C). The fibrosis-associated terms were similar to those in RV tissues, and there were no terms with fibrotic production (Figs. 3C and 5D).

Similar to RV tissue results, the data showed that hypertrophy maker genes (Nppa) were highly upregulated in the PO-RV cardiomyocytes, while fibrosis-related maker genes (COL3a1) and myofibroblast marker (Acta2) showed no changes in the PO-RV cardiomyocytes (Figs. 5E–F). In contrast to RV tissue results, there were no changes in the expression of NPPB in the PO-RV cardiomyocytes; and there was upregulated expression of COL1a1 and COL1a2 in the PO-RV cardiomyocytes (Figs. 5E–F).

**Fig. 6:**
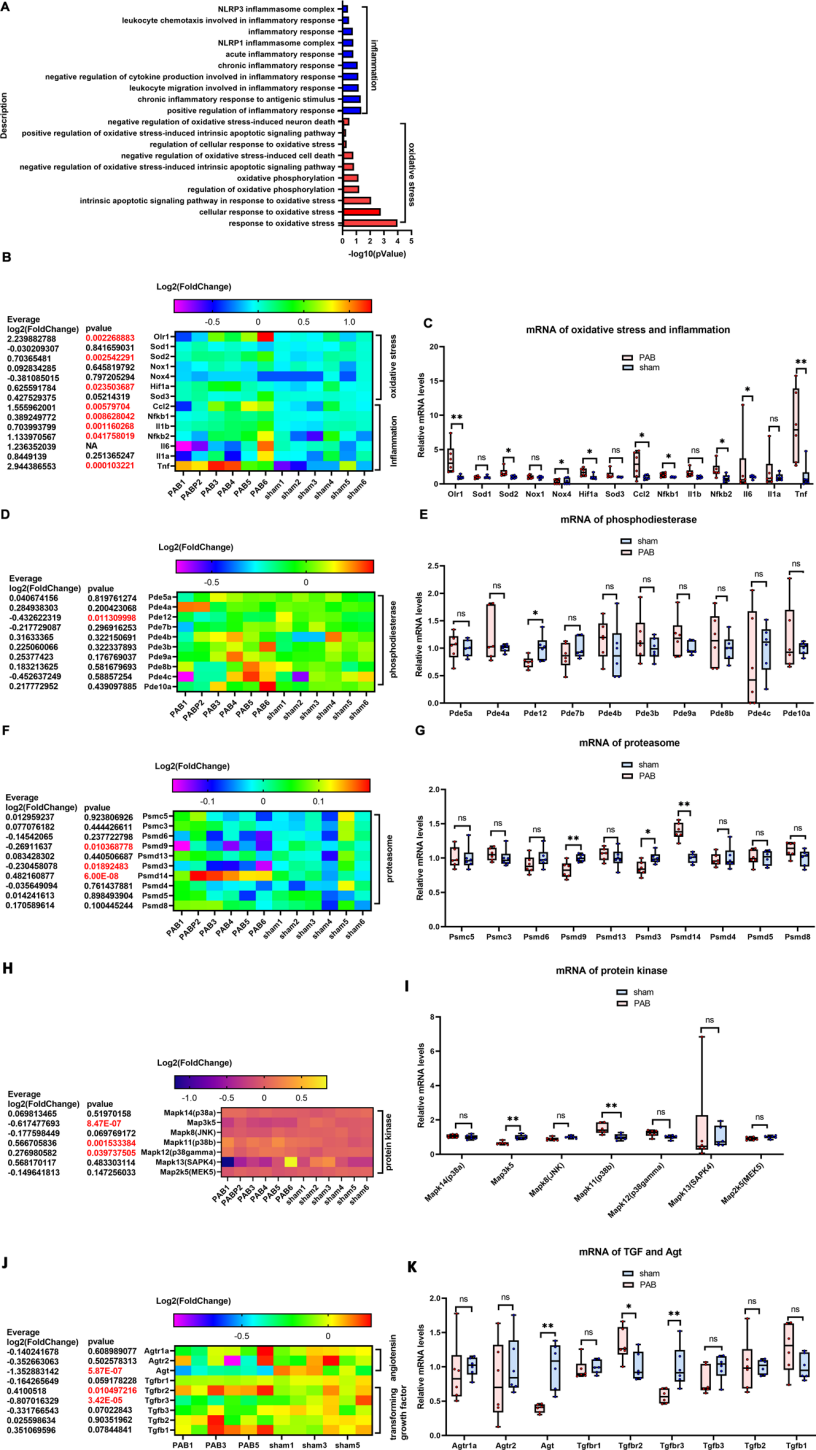
Bulk RNA-seq analysis of RV cardiomyocytes demonstrates that the signaling pathways that account for hypertrophy and fibrosis in neonatal RV are different from those in adult RV. (**A**) Top 10 enriched GO terms associated with oxidative stress and inflammation in upregulated DEGs in cardiomyocytes. (**B**) Heatmap of Log2 (fold-change) of oxidative stress and inflammation marker genes. (**C**) Relative mRNA levels of oxidative stress and inflammation marker genes. (**D**) Heatmap of Log2 (fold-change) of phosphodiesterase genes. (**E**) Relative mRNA levels of phosphodiesterase genes. (**F**) Heatmap of Log2 (fold-change) of proteasome genes. (**G**) Relative mRNA levels of proteasome genes. (**H**) Heatmap of Log2 (fold-change) of protein kinase genes in cardiomyocytes. (**I**) Relative mRNA levels of protein kinase genes in cardiomyocytes. (**J**) Heatmap of Log2 (fold-change) of TGF and AGT in cardiomyocytes. (**K**) Relative mRNA levels of TGF and AGT genes in cardiomyocytes. Cluster heatmap was generated using the OECloud tools (v1.26) at https://cloud.oebiotech.com.

There was a similar enrichment of GO terms associated with inflammation and oxidative stress in PO-RV cardiomyocytes, as seen in PO-RV tissues (Fig. 6A). As in PO-RV tissues, the expression of OLR1 and HIF1a was upregulated in PO-RV cardiomyocytes (Figs. 6B–C). Contrary to the RV tissue results, the expression of SOD2, CCl2, NFKB1, NFKB2, IL1b, and TNF was upregulated (Figs. 6B–C). The other key genes, such as SOD1, SOD3, NOX1, NOX4, IL6, and IL1a, were unchanged (Figs. 6B and C).

Similar to the PO-RV tissue results, the expression of all kinds of phosphodiesterases was unchanged, except for PDE12, which was downregulated in PO-RV cardiomyocytes (Figs. 6D and E); the expression of proteasomes in PO-RV cardiomyocytes was downregulated or unchanged, except for PSMD 14, which was upregulated (Figs. 6F and G), similar to PO-RV tissue results; the expression of most protein kinases in PO-RV cardiomyocytes was downregulated or unchanged, except for MAPK11 and MAPK12, which were upregulated, similar to PO-RV tissue results (Figs. 6H and I); and most of the AGTs and TGFs in PO-RV cardiomyocytes were downregulated or unchanged, except for TGFB2, which was upregulated, similar to PO-RV tissue results (Figs. 6J and K).

### A unique hypertrophic and fibrotic feature of neonatal RV in response to RVPO, as revealed by single-cell RNA-seq analysis

**Fig. 7:**
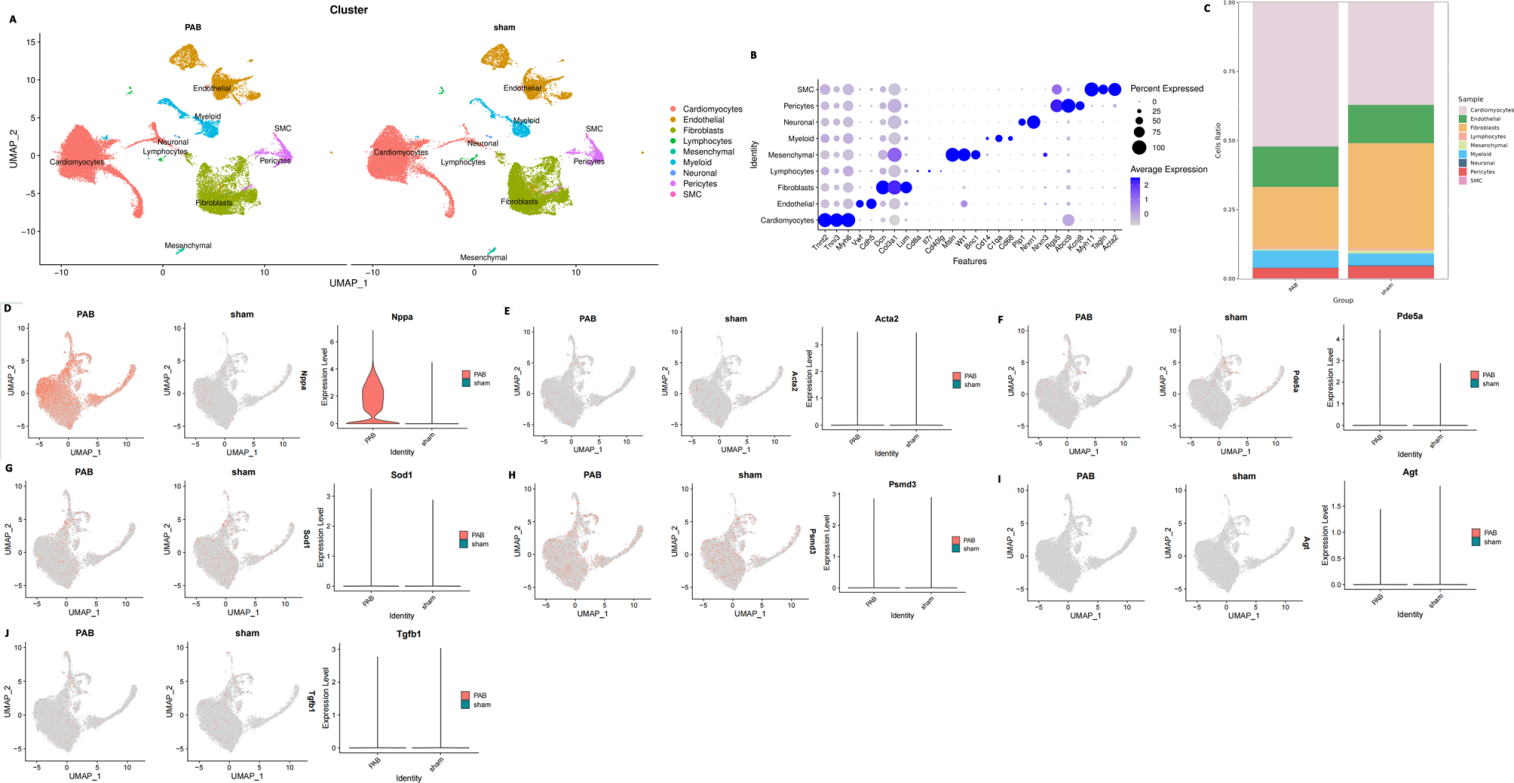
Single-cell RNA-seq analysis of RV tissues demonstrates that the signaling pathways that account for hypertrophy and fibrosis in neonatal RV are different from those in adult RV. (**A**) Left panel: Umap analysis demonstrates that there are mainly 9 types of cells in the neonatal PO-RV. (**B**) The expression levels of marker genes in each cell type. (**C**) Percentage of each cell type in normal (sham) and PO (PAB)-RV. (**D**) Umap analysis demonstrates that the expression of Nppa (maker of hypertrophy) is significantly increased in PO (PAB)-cardiomyocytes than in sham (normal) cardiomyocytes. (**E**) Umap analysis demonstrates that the expression of Acta2 (maker of fibrosis) in PO (PAB)-cardiomyocytes is not different from that in sham (normal) cardiomyocytes. (**F**) Umap analysis demonstrates that the expression of PDE5a in PO (PAB)-cardiomyocytes is not different from that in sham (normal) cardiomyocytes. (**G**) Umap analysis demonstrates that the expression of SOD1 in PO (PAB)-cardiomyocytes is not different from that in sham (normal) cardiomyocytes. (**H**) Umap analysis demonstrates that the expression of Psmd3 in PO (PAB)-cardiomyocytes is not different from that in sham (normal) cardiomyocytes. (**I**) Umap analysis demonstrates that the expression of AGT in PO (PAB)-cardiomyocytes is not different from that in sham (normal) cardiomyocytes. (**J**) Umap analysis demonstrates that the expression of AGT in PO (PAB)-cardiomyocytes is not different from that in sham (normal) cardiomyocytes.

To further validate the RNA-seq results and explore possible communication between different cell types, single-cell RNA-seq was performed on neonatal PO-RV, documenting this analysis of neonatal PO-RV for the first time. The results showed that the percentage of cardiomyocytes increased in the neonatal PO-RV (Figs. 7A–C) with a higher expression of hypertrophy marker Nppa (Fig. 7D) and no differences in the fibrotic marker Acta2 (Fig. 7D).The results also showed that there were no differences in the expression of PED5a, PSMC3, AGT, and TGF(Figs. 7E–J).

## Discussion

This study first demonstrates the unique feature of neonatal RV hypertrophy and fibrosis—a transition stage between normal and dysfunctional RV, using previously reported neonatal RVPO models induced by microsurgery^11,30^. The data show that many genes or pathways of hypertrophy and fibrosis that are upregulated in adult RVPO models are unchanged or downregulated in neonatal RVPO models. These results partly explain why adult HF treatment-effective drugs, such as sildenafil or enalapril, fail to treat pediatric HF.While temporal alignment between neonatal and adult RVPO studies presents methodological challenges, three key differences emerge when using HF as an endpoint: (1) Neonatal animals progress to failure significantly faster than adults; (2) Fibrotic remodeling is minimal or absent in neonates despite severe dysfunction; and (3) The underlying pathophysiology appears distinct, likely reflecting developmental differences in metabolic adaptation and stress response pathways. More importantly, to the best of our knowledge, this is the first study to document single-cell RNA-sequence data of neonatal PO-RV, providing an importance reference for future basic or clinical studies, which might help to advance pediatric HF treatment.

Most of the PO-RV cardiomyocytes results were similar to PO-RV tissue results. The differences were in the expression of SOD2, SOD3, IL-1b, CCl2, NFKB1, NFKB2, TNF, PDE12, MAPK11, MAPK12, PSMD8, PSMD14, TGFB2, TGFB3, TGFBR3, and AGT. This may be because these proteins are produced by cardiomyocytes and act on other cells besides cardiomyocytes. For example, SOD3 is secreted by endothelial cells and exists in the extracellular matrix (ECM)^41,42^. In addition, The disparity in Col1-3/Acta2 expression between RV tissue and cardiomyocytes likely reflects cell-type-specific responses to RVPO. While tissue-level markers integrate contributions from fibroblasts (potentially suppressed by compensatory mechanisms), cardiomyocytes may autonomously upregulate ECM genes under mechanical stress. This aligns with reports of cardiomyocyte-endowed fibrogenic potential under pathological conditions^43^. Further studies profiling fibroblast-specific markers are warranted.

However, this is a descriptive study with many interesting phenomena and mechanisms yet to be explored, such as why RVPO does not induce neonatal cardiac fibrosis as seen in adult animal models. Inflammation and oxidative stress are two of the initiators of cardiac hypertrophy and fibrosis^15,16^. While RVPO induced enrichment of inflammation and oxidative stress in neonatal RVs (Figs. 3A and 5A), unlike adult animals, neonatal RV mainly induces the enrichment of anti-inflammatory and antioxidant genes (Figs. 3A and 5A). As shown in Fig. 3A, SOD3 was one of the most upregulated genes in neonatal PO-RV. It has been shown that SOD3 is critical in protecting the heart against PO-induced hypertrophy and fibrosis in the left ventricle^41,42^. However, multiple clinical studies have demonstrated that SOD3 was downregulated in failing adult hearts^41^. Thus, future studies should focus on SOD3 to show that the upregulation of SOD3 in neonatal RV is one of the underlying mechanisms that protects neonatal RV from fibrosis.

Another important question is why neonatal RVs unavoidably develop hypertrophy under conditions of PO. These results show that the average life expectancy of a neonatal rat or mouse with PAB surgery is 21–30 days^11^. However, when de-banding surgery was performed on P14, the survival rate and time of the RVPO in rats/mice were increased with decreased cardiac hypertrophy^11^, suggesting a high recovering ability of neonatal RVs. These results suggest that the first choice for treating neonatal RV hypertrophy and HF may be to promptly address the causes of RV hypertrophy, such as PAH or RV outflow tract obstruction. However, PAH or some type of RV outflow tract obstruction is generally hard to address. Under RVPO conditions, finding treatment targets may be the second choice. Study data show that the NLRP3 inflammasome complex was among the top 10 enrichment terms for upregulated DEGs in both RV tissues and RV cardiomyocyte RNA-seq analysis (Figs. 3A and 5A). In recent years, the NLRP3 inflammasome complex has been recognized as playing a critical role in cardiovascular diseases, but not in neonatal RVPO models^44,45^. Thus, the NLRP3 inflammasome complex should be the focus of studies on anti-neonatal RV hypertrophy induced by RVPO.

In summary, this observational study screened a range of key genes and pathways known to play critical roles in adult RVPO models within neonatal RVPO models and identified the unique hypertrophic and fibrotic feature of neonatal RV in response to PO, which helps to explain why adult-effective anti-HF drugs fail to treat pediatric HF. More importantly, this study documented the first single-cell RNA-seq data of neonatal PO-RV, providing an important reference for future basic or clinical investigations on pediatric RV failure.

## Electronic supplementary material

Below is the link to the electronic supplementary material.

Supplementary Material 1

## Data Availability

All of the bulk RNA-seq data were deposited in the GEO database (https://www.ncbi.nlm.nih.gov/geo) with accession numbers GSE139561 and GSE232054. Single-cell RNA-seq data are available from the corresponding author upon reasonable request.Information on all primers is provided in Supplemental Tables S1.

## Abbreviations

PAB: Pulmonary artery banding
GO: Gene Ontology
KEGG: Kyoto Encyclopedia of Genes and Genomes
HF: Heart failure
RNA-seq: RNA sequencing
PA: Pulmonary artery
H&E: Hematoxylin and eosin
FPKM: Fragments Per Kilobase of transcript sequence per Million
TOF: Tetralogy of Fallot
PBS: Phosphate buffer saline
DAPI: 4’,6-diamidino-2-phenylindole
pH3: Phospho-histone H3
RV: Right ventricle
RVCM: Right ventricular cardiomyocyte
