# Supplementary material for: The unique hypertrophic and fibrotic features of neonatal right ventricle in response to pressure overload
Source: Sci Rep. 2025 May 20;15:17525. doi: 10.1038/s41598-025-01427-y (PMC12092643; doi:10.1038/s41598-025-01427-y)
Supplement: Supplementary file 1 — Supplementary Material 1 [file 41598_2025_1427_MOESM1_ESM.docx]

**Supplemental Table S1 Primer information**

| Gene (mouse) |  | Sequence (5'->3') |
| --- | --- | --- |
| *Nppa* | Forward | GCTTCCAGGCCATATTGGAG |
|  | Reverse | GGGGGCATGACCTCATCTT |
| *Nppb* | Forward | GAGGTCACTCCTATCCTCTGG |
|  | Reverse | GCCATTTCCTCCGACTTTTCTC |
| *Olr1* | Forward | CAGTGGGACACCCGACATC |
|  | Reverse | GTACACGTTTCCAAACTTGCC |
| *Sod1* | Forward | AACCAGTTGTGTTGTCAGGAC |
|  | Reverse | CCACCATGTTTCTTAGAGTGAGG |
| *Sod2* | Forward | CAGACCTGCCTTACGACTATGG |
|  | Reverse | CTCGGTGGCGTTGAGATTGTT |
| *Sod3* | Forward | CCTTCTTGTTCTACGGCTTGC |
|  | Reverse | TCGCCTATCTTCTCAACCAGG |
| *Nox1* | Forward | GGTTGGGGCTGAACATTTTTC |
|  | Reverse | TCGACACACAGGAATCAGGAT |
| *Nox4* | Forward | GAAGGGGTTAAACACCTCTGC |
|  | Reverse | ATGCTCTGCTTAAACACAATCCT |
| *Tgfbr1* | Forward | TCTGCATTGCACTTATGCTGA |
|  | Reverse | AAAGGGCGATCTAGTGATGGA |
| *Tgfbr2* | Forward | CCGCTGCATATCGTCCTGTG |
|  | Reverse | AGTGGATGGATGGTCCTATTACA |
| *Tgfbr3* | Forward | GGTGTGAACTGTCACCGATCA |
|  | Reverse | GTTTAGGATGTGAACCTCCCTTG |
| *Tgfb3* | Forward | CCTGGCCCTGCTGAACTTG |
|  | Reverse | TTGATGTGGCCGAAGTCCAAC |
| *Tgfb2* | Forward | CTTCGACGTGACAGACGCT |
|  | Reverse | GCAGGGGCAGTGTAAACTTATT |
| *Tgfb1* | Forward | ATGTCACGGTTAGGGGCTC |
|  | Reverse | GGCTTGCATACTGTGCTGTATAG |
| *Col1a1* | Forward | GCTCCTCTTAGGGGCCACT |
|  | Reverse | CCACGTCTCACCATTGGGG |
| *Col1a2* | Forward | GTAACTTCGTGCCTAGCAACA |
|  | Reverse | CCTTTGTCAGAATACTGAGCAGC |
| *Col3a1* | Forward | CTGTAACATGGAAACTGGGGAAA |
|  | Reverse | CCATAGCTGAACTGAAAACCACC |
| *Acta2* | Forward | GTCCCAGACATCAGGGAGTAA |
|  | Reverse | TCGGATACTTCAGCGTCAGGA |
| *Agtr1a* | Forward | AACAGCTTGGTGGTGATCGTC |
|  | Reverse | CATAGCGGTATAGACAGCCCA |
| *Agtr2* | Forward | AACTGGCACCAATGAGTCCG |
|  | Reverse | CCAAAAGGAGTAAGTCAGCCAAG |
| *Agtr1b* | Forward | TGGCTTGGCTAGTTTGCCG |
|  | Reverse | ACCCAGTCCAATGGGGAGT |
| *Agt* | Forward | TCTCCTTTACCACAACAAGAGCA |
|  | Reverse | CTTCTCATTCACAGGGGAGGT |
| *Pde5a* | Forward | CGGCCTACCTGGCATTCTG |
|  | Reverse | GCAAGGTCAAGTAACACCTGATT |
| *Pde4a* | Forward | GAACCGGGAACTCACACACC |
|  | Reverse | GTACTCTGAGACCTGGTTTCCT |
| *Pde12* | Forward | ATGTGCTCAATGTGGACGC |
|  | Reverse | GGGAAGCCAGCCATGATGTAG |
| *Pde7b* | Forward | TGCTAGGAGATGTACGACTAAGG |
|  | Reverse | GGGCCTGCGGTATAATCCC |
| *Pde4b* | Forward | CGCAGGGAGTCGTTCCTCTA |
|  | Reverse | CTCCTGTGGTCGCACACTTG |
| *Pde3b* | Forward | AAAGCGCAGCCGGTTACTAT |
|  | Reverse | CACCACTGCTTCAAGTCCCAG |
| *Pde9a* | Forward | CCACCATCTCCCTTTTAACCAC |
|  | Reverse | CAGCACGCCCTGGATAAGT |
| *Pde8b* | Forward | AGAGCGGTGTGATCTACTGC |
|  | Reverse | CGTCGGTCTGCACGAAGAG |
| *Pde4c* | Forward | AGCTTTGACCTCGAAAATGGG |
|  | Reverse | GTCCGAACGGTACAGGAAGG |
| *Pde10a* | Forward | GGACAGAGACAAGCGAGATGA |
|  | Reverse | GGTGTGCTCTTGCTAGGCG |
| *Psmc5* | Forward | AACTTGACCAGGGGGATCAAC |
|  | Reverse | AGTCCTCCTGAGTGACGTGG |
| *Psmc3* | Forward | GACCGTGTGGGATGAAGCTG |
|  | Reverse | CGCTGGACAATCTCTTCCGTG |
| *Psmd6* | Forward | ATGCCGCTAGAAAACCTAGAAGA |
|  | Reverse | GTCGAGGGATTTACACAAGGC |
| *Psmd9* | Forward | ATGTCGGGTGAAGACGTTCC |
|  | Reverse | GGTTCGTTCATGCCAATTCCTT |
| *Psmd13* | Forward | TTTGGCACCGTTTGGAAGAG |
|  | Reverse | TACCAGGGACAGAGGGTTTAC |
| *Psmd3* | Forward | CCGGGAGGGGAACAAGAAC |
|  | Reverse | AGCCTTCTCTAGCTGTCTCAC |
| *Psmd14* | Forward | TGATGATTACACCGTCAGAGTGA |
|  | Reverse | GGAACACTGGATCAACTGCTT |
| *Psmd4* | Forward | GACCCGGAGTTCCTTCAGAG |
|  | Reverse | CAGAGCCCCCATGACACTTC |
| *Psmd5* | Forward | CGCTGTTCTCCTTGCTTAACC |
|  | Reverse | GTCATCAGGGTGAGTCAGTCC |
| *Psmd8* | Forward | GGCATGTACGAGCAACTCAAG |
|  | Reverse | GCTCAAGCAGAACCAACTTCA |
| *Mapk14(p38a)* | Forward | TGACCCTTATGACCAGTCCTTT |
|  | Reverse | GTCAGGCTCTTCCACTCATCTAT |
| *Map3k5* | Forward | GTTTCTGGAACGTGGAGAGC |
|  | Reverse | CTTCCCGAAATGCAGGGTC |
| *Mapk8(JNK)* | Forward | AGCAGAAGCAAACGTGACAAC |
|  | Reverse | GCTGCACACACTATTCCTTGAG |
| *Mapk11(p38b)* | Forward | GCGGGATTCTACCGGCAAG |
|  | Reverse | GAGCAGACTGAGCCGTAGG |
| *Mapk12(p38gamma)* | Forward | AAGGGCTTTTACCGCCAGG |
|  | Reverse | GGCGCAACTCTCTGTAGGC |
| *Mapk13(SAPK4)* | Forward | ATGAGCCTCACTCGGAAAAGG |
|  | Reverse | GCATGTGCTTCAAGAGCAGAA |
| *Mapk7(ERK5)* | Forward | GTGGGGGACGAGTACGAGAT |
|  | Reverse | TGGTCACCACATCAAAAGCAT |
| *Map2k5(MEK5)* | Forward | AAGCAGCCCAAGGAGAGAC |
|  | Reverse | GAACTGCACGATGAATGGGTG |
| *Ccl2* | Forward | TTAAAAACCTGGATCGGAACCAA |
|  | Reverse | GCATTAGCTTCAGATTTACGGGT |
| *Nfkb1* | Forward | ATGGCAGACGATGATCCCTAC |
|  | Reverse | TGTTGACAGTGGTATTTCTGGTG |
| *Nfkb2* | Forward | GGCCGGAAGACCTATCCTACT |
|  | Reverse | CTACAGACACAGCGCACACT |
| *Il6* | Forward | TAGTCCTTCCTACCCCAATTTCC |
|  | Reverse | TTGGTCCTTAGCCACTCCTTC |
| *Il1b* | Forward | GCAACTGTTCCTGAACTCAACT |
|  | Reverse | ATCTTTTGGGGTCCGTCAACT |
| *Il1a* | Forward | CGAAGACTACAGTTCTGCCATT |
|  | Reverse | GACGTTTCAGAGGTTCTCAGAG |
| *Tnf* | Forward | CCCTCACACTCAGATCATCTTCT |
|  | Reverse | GCTACGACGTGGGCTACAG |
|  |  |  |
| Gene (rat) |  | Sequence (5'->3') |
| *Nppa* | Forward | GATCTGCCCTCTTGAAAAGC |
|  | Reverse | GCCCTGTATTCTAATTTCCC |
| *Nppb* | Forward | GTATGTGTGTGTGTGTCTGAGG |
|  | Reverse | GCTGTGCAAGTATGTCTGAG |
| *Olr1* | Forward | GAAGGAAGAGGTCTGGAAAG |
|  | Reverse | CAAGACATTGGGGACATTTGC |
| *Sod1* | Forward | AACCAGTTGTGTTGTCAGGAC |
|  | Reverse | CTCTGTACACCCTATACCTCCT |
| *Sod2* | Forward | CACAAGCAACACTACACTGG |
|  | Reverse | CTCGGTGGCGTTGAGATTGTT |
| *Sod3* | Forward | AAGGTGTGTGTGTGTGTAGG |
|  | Reverse | TGTGTGTGTGTGTGTGTGTGTG |
| *Nox1* | Forward | CAGGGGACTGGACAGAAAAT |
|  | Reverse | GAAGGAAGCAAAGGGAGTGA |
| *Nox4* | Forward | GCCTATACTGTGCTGAGAGA |
|  | Reverse | GCTTGATGGAGGCAGTAGTA |
| *Tgfbr1* | Forward | GATTCTATCTTGGGAAGGGC |
|  | Reverse | CCCCCCAAGAATATGATCAC |
| *Tgfbr2* | Forward | TCAAGATCTTCCCCTACGAG |
|  | Reverse | CACAGGCAACAGGTCAAATC |
| *Tgfbr3* | Forward | TCCAGACCAATGGCTACTCA |
|  | Reverse | GTTTAGGATGTGAACCTCCCTTG |
| *Tgfb3* | Forward | GGAGTGGACAACGAAGATGA |
|  | Reverse | GGGTTCAGGGTGTTGTATAG |
| *Tgfb2* | Forward | CTGCATCTCACCTACTTCCT |
|  | Reverse | TCCTTCCCCCTGGCTTATTT |
| *Tgfb1* | Forward | CCTGAGTGGCTGTCTTTTGA |
|  | Reverse | GACAGAAGTTGGCATGGTAG |
| *Col1a1* | Forward | TCTCAAGATGGTGGCCGTTA |
|  | Reverse | GTTGCAGTAGACCTTGATGG |
| *Col1a2* | Forward | TGAACGTGGTGAGGTTGGTC |
|  | Reverse | GCCCTTTGGTTCCCTTTTCT |
| *Col3a1* | Forward | TGTACATTGGTTTTAGGGTC |
|  | Reverse | ATAGGGAGGTTGTGGAGAAG |
| *Acta2* | Forward | TCACACCCTTGCTGCACAAA |
|  | Reverse | CTACACCTCTGTCCCTTGAA |
| *Agtr1a* | Forward | TAATGACCCATCTCTCCAGC |
|  | Reverse | GCTACCACTACCCTCACATT |
| *Agtr2* | Forward | ATTTACTCCCCCCAGAACAG |
|  | Reverse | CCACCTCTCATGCTCTATCA |
| *Agtr1b* | Forward | CCATCACCATCTGCATAGCT |
|  | Reverse | AACCCTCTCACTCCACTTCA |
| *Agt* | Forward | GTGACAGGGTGGAAGATGAA |
|  | Reverse | CTTGGAAGTGAACGTAGGTG |
| *Pde5a* | Forward | GTGGGAGGAAGTAGGGAAAT |
|  | Reverse | CCAAGCCACAGAAGATGACA |
| *Pde4a* | Forward | AGGAAGAGGAGGAAGAGGAT |
|  | Reverse | GTAGAGATGATGGCAGAATTGTC |
| *Pde12* | Forward | AGGATCATGAAGACTGGGCT |
|  | Reverse | CTGGTGGGTGGTAACTTCTT |
| *Pde7b* | Forward | TAACCACCACCTTGCCAACT |
|  | Reverse | GACTCTTTCACTCCACTGCT |
| *Pde4b* | Forward | GGAGAGGGGAATGGAGATTA |
|  | Reverse | GTGCTGCTGAAATAGTTGGG |
| *Pde3b* | Forward | GACCAATTCCTGGCTTACAG |
|  | Reverse | GGAGGAAGTTGTATTCTGGG |
| *Pde9a* | Forward | AGCAACCCCTTCCACAACTT |
|  | Reverse | ATCCATAATTTCAGCGTGCC |
| *Pde8b* | Forward | ATGGCTTGAGGAGACTGTCA |
|  | Reverse | GTGGAGTTGTGGTAGGCATT |
| *Pde4c* | Forward | GCTAAGGAGCTTGAAGACAC |
|  | Reverse | GAGAAACTGGTTGGAGACAC |
| *Pde10a* | Forward | CCATGGAGAAGCTGTCTTAC |
|  | Reverse | CACAGACAGGCAATTAGCAG |
| *Psmc5* | Forward | CCAGACTCAACCTACGAGAT |
|  | Reverse | ACAGCTCCCTCACCATTCTT |
| *Psmc3* | Forward | TGCCCACCGAATATGACTCT |
|  | Reverse | CCCATACATCAGCACTCCTT |
| *Psmd6* | Forward | GACCTCATCACTCGAAACAC |
|  | Reverse | CACTTCAAGAATCTCTGCCC |
| *Psmd9* | Forward | CGGGCAGATGTGGATTTGTA |
|  | Reverse | GAGACTGGAAGTTTTGGGTG |
| *Psmd13* | Forward | CAGAACAGCAAGAAAGAGCC |
|  | Reverse | GAGAGTGCCTTCATCACCAA |
| *Psmd3* | Forward | AGCTAGTGTCCAAGTCTGTG |
|  | Reverse | TTGGGTGAGAAGGAAGTAGG |
| *Psmd14* | Forward | GGACATCAACACTCAGCAGA |
|  | Reverse | GTCAATCCTTCCATCCAACTC |
| *Psmd4* | Forward | ATTTTGGGGAAGAGGAGGTG |
|  | Reverse | CATCTTCAGTAGGGCATCATC |
| *Psmd5* | Forward | TGATTGGTGTGGCTGTAGAC |
|  | Reverse | GGTGATAGGTACAGAAGAGAGG |
| *Psmd8* | Forward | TCAAACACCCTGTATCCCTC |
|  | Reverse | GCATCCTGCAATTTCATCCC |
| *Mapk14(p38a)* | Forward | CCTTAGGGATGTGTGTGTGT |
|  | Reverse | GTGATATGGTAAAGGCAGGG |
| *Map3k5* | Forward | GAAGGAGACTGTGAAGGTGA |
|  | Reverse | CATTTTGAACGGAGGAGAGC |
| *Mapk8(JNK)* | Forward | TGCAATACCCAGGCACCTTT |
|  | Reverse | TACAACCCTGCAGCTCATAC |
| *Mapk11(p38b)* | Forward | GAGGAGATGACTGGATATGTGG |
|  | Reverse | GGCAGAGACTGGATGTATGT |
| *Mapk12(p38gamma)* | Forward | GTGGACATTTGGTCTGTTGG |
|  | Reverse | GAAGGGACTCAAAGTATGGG |
| *Mapk13(SAPK4)* | Forward | GATGACTGGCTATGTGGTGA |
|  | Reverse | GAAGGGTTCAAAGAAGGGGT |
| *Mapk7(ERK5)* | Forward | GTGGCTGAGATTGAGGACTT |
|  | Reverse | GAGGGCTGCTTTGGTATTGT |
| *Map2k5(MEK5)* | Forward | CGGCCAGATGAATGAACAAG |
|  | Reverse | TATACATCCAAAGACCCCCC |
| *Ccl2* | Forward | GTGTCCCAAAGAAGCTGTAG |
|  | Reverse | GGTTGTGGAAAAGAGAGTGG |
| *Nfkb1* | Forward | GGATGTGGGGTTTCAGGATA |
|  | Reverse | GAAGGTGGATGATGGCTAAG |
| *Nfkb2* | Forward | TTTCTCCTCCTTCGCCTACA |
|  | Reverse | GCTATCTGCTCAATGACACC |
| *Il6* | Forward | AATCTGCTCTGGTCTTCTGG |
|  | Reverse | TGAGTTGGATGGTCTTGGTC |
| *Il1b* | Forward | AAGGAGAGACAAGCAACGAC |
|  | Reverse | GGGGAAGGCATTAGGAATAG |
| *Il1a* | Forward | CCTGTGTTGCTGAAGGAGAT |
|  | Reverse | GTATCATATGTCGGGCTGGT |
| *Tnf* | Forward | CATACCAGGAGAAAGTCAGC |
|  | Reverse | AGAGTAATGGGGGTCAGAGT |
